# Dirt, Saliva and Leprosy: Anti-Inflammatory and Anti-Infectious Effects

**DOI:** 10.3390/diseases7010031

**Published:** 2019-03-22

**Authors:** Helieh S. Oz

**Affiliations:** Department of Medicine, University of Kentucky Medical Center, Lexington, KY 40536, USA; Hoz2@email.uky.edu

**Keywords:** soil, saliva, leprosy, Hansen’s disease, biomarkers

## Abstract

Ancient Egyptians smeared a mixture of dark soil on their eyelids and believed it protected eyes from unknown forces (illness). Recent studies have proven that the dark soil across the Nile River is rich in natural compounds including lead sulfide, which in low levels, promotes the production of nitric oxide (240-fold) by keratinocytes, with strong immune stimulatory and antimicrobial properties. Current investigations reveal anti-inflammatory and anti-infectious activities—including cytokines and chemokines—in saliva, as well as its friendly microbiota, which lines the surface of the oral cavity, its protection against inflammatory and infectious organisms in the stoma and other organs, such as the cardiovascular and central nervous systems. In fact, saliva may soon become a safe and practical surrogate biomarker for genomic/proteomic evaluations and to replace painful blood drawing and its side effects. Another example is leprosy, or Hansen’s disease, a chronic inflammatory syndrome and neglected tropical disease, which affects the skin, and peripheral and trigeminal neurons causing a lack of sensation to heat and cold and loss of extremities. Leprosy has horrified humans for over 2000 years, as lepers were considered unclean sinners and were subsequently drawn out of towns. This communication scrutinizes the past and the present state of saliva and leprosy to encounter possible mystery and/or wisdom in ancient healing as the mixture of “sputum and dirt” as reported in the biblical time.

## 1. Introduction and Conclusion

Cleopatra’s darkened eyelids and eyebrows were primarily to protect her eyes from desert sunburn and common infections than being exclusively used as cosmetics. As the dark Egyptian soil across the Nile River is reported to be rich in natural compounds and minerals, in addition to Galena and lead sulfide (PbS) [1,2]. Though in high doses, lead is toxic, in low levels it can: Promote the production of nitric oxide, about 240-fold, by skin cells (keratinocytes); present anti-microbial properties to activate macrophages; increase blood flow; and provoke immune responses in order to fend off infections. Ancient Egyptians smeared a mixture of dark soil and plants called “kohl” on their eyelids, believing that it would protect their eyes from unknown forces and illnesses (like infections and conjunctivitis). “Indeed, this seemingly basic beauty product could have been one of the most archaic ophthalmological preparations ever known by man” [3,4].

Recent studies reveal that the anti-inflammatory and anti-infectious activities in saliva could protect the oral cavity from inflammatory and infectious agents [5,6,7]. In addition, the friendly microbiota, which lines the surface of the mucosal cavity, counter infectious organisms from seeding in the stoma and other organs, such as the cardiovascular and even the central nervous system. Besides, the powerful cytokines/chemokines, with anti-inflammatory mediators and anti-infectious activity in the saliva, can protect body entrance, which is open to all sorts of nutrients, as well as toxins and infectious agents [8,9,10]. In fact, salvia may soon become a safe and a practical surrogate biomarker for genomic/proteomic evaluations, as well as replacing painful blood drawing procedures for routine and reliable analysis with no possible transmission of blood-borne diseases, such as HIV/AID and viral hepatitis when used for diagnostic purposes. Additionally, saliva is being increasingly acknowledged as having diagnostic values with regard to genetic biomarkers [11]. A recent pilot clinical trial explored huntingtin protein in saliva as a safe and non-invasive tool to diagnose Huntington disease in patients with the early stage of the disease [12].

As in the biblical story, Jesus of Nazareth in the land of Egypt “spitted on the ground” and made a paste to cover the eyes of a blind man who possibly had infected and swollen eyes, covered with inflammation, and pus of germs. Indeed, this may support and explain that Jesus, a healer of his time, had knowledge of infectious and inflammatory diseases and a possible cure for such.

Another example is leprosy, or Hansen’s disease, a chronic inflammatory syndrome and neglected tropical disease, caused by slow-growing Gram-positive bacillus *Mycobacterium leprae*, which was discovered by Armauer Hansen, MD (1873) from his patient with lesions. Leprosy is transmitted possibly through cough, sneeze and open wounds and prolonged contact with a patient. Leprosy affects the skin and peripheral and trigeminal neurons, which results in a lack of sensation to heat and cold, and the loss of extremities. Eye complications include keratitis, corneal ulceration, conjunctivitis, cataract and blindness [13]. The weakness of the eyelids, and the lack of the lid’s proper closure to protect the eyes, can eventually lead to blindness. The World Health Organization (WHO) has classified leprosy according to the number of skin injuries, and when six lesions or more are present it is called multibacillary. Otherwise, it is called paucibacillary if the patient suffers from five or fewer lesions [14]. Patients with exaggerated immune response who develop paucibacillary leprosy are mostly non-infectious, but those with a low immune response develop multibacillary leprosy with multiple lesions, which can become infectious. Overall, lepers have a dry mouth, low saliva, and a loss of function in the soft and hard palate tissues, resulting in periodontitis and loss of teeth [15]. Most patients with lesions in the oral cavity are affected with the multibacillary form. Leprosy has horrified humans throughout the ages from biblical times (>2000 years) where lepers were considered as unclean sinners and drawn out of towns. Indeed, the chance to become infected with bacillus is extremely low, as only 5% of the global population is susceptible to *M. leprae* [14]. According to the Center for Disease Control and Prevention (CDC), about 150 people in the U.S.A, mostly in the Southern States, are diagnosed with leprosy each year [16]. The rate is highest especially in India (58%), Brazil (16%), South East Asia and Africa. The WHO reports a total of 200,000 people are infected globally each year [14]. Armadillos are the only non-human animals found to be infectious in nature and serve as a source of infection, specifically in the Southern States [16]. In addition, asymptomatic carriers may release bacilli from the nasal cavity to contaminate the surrounding milieu, and *M. leprae* has been isolated from the soil.

The last time a lepers’ colony existed in the mainland U.S.A, was in the Leper Home in Louisiana, established in 1894 and renamed the National Leprosarium in Carville in 1917, which located between Baton Rouge and New Orleans. Indeed, the lepers’ colony served as an educational source for infectious diseases students and residents, with profound, unforgettable and lasting effects on those who had visited (author’s personal experience, over 4 decades ago). It closed in 1999 due to the lack of funding and later transformed into a historical museum for leprosy. Currently, the outpatient leprosy clinic is operating in Baton Rouge, L.A. At present, there is only one Leper’s colony left in the U.S.A, Kalaupapa in Hawaii, on the island of Molokai, with six elderly residents who have voluntarily stayed behind due to major deformations and fear of rejection by society. Children with leprosy exhibit significant decreases in the salivary total antioxidant capacity compared to healthy controls (*p* < 0.001) [17]. In the early stages of the disease, multi-antibiotic therapy (rifampicin, dapsone and clofazimine), which has been used since 1982, can cure patients [14]. Although therapy is proven to protect and be effective when prescribed before nerve injuries, if not treated, then the symptoms will advance to deformation and loss of extremities, such as the fingers, toes, nose, ears and lips, as well as blindness, with the possibility of irreversible damage. The patients, who suffer from exaggerated hyperactive immune responses, which are generated against very low numbers of bacterial loads, can create an impotent confounding factor for the management and treatment of leprosy [18]. Yet, Jesus had no fear to be near the lepers, nor to speak or touch them or being touched by lepers. It was not until the last century when early clinical studies (1922) showed spontaneous inactivation or self-healing in some leper populations, specifically children, who were followed up less than one year [19]. Subsequent trials lasting for over 25 years confirmed that some pediatric populations developed only minor or no symptoms. Even some patients with early lesions of leprosy may experience their infection become stabilized, or even experience their lesions disappear entirely due to effective immune responses, which trigger self-healing [20,21]. In South India, 11% of those with paucibacillary [22] and 78% of infected children from Culion Island in the Philippines [23] showed self-recovery, thus demonstrating that leprosy is not a highly contagious disease. These findings further proved that leprosy occurs only in a limited number of people, possibly in those with immunity predisposed to the disease. It is hypothesized that the hypothiocyanite anion, with antimicrobial oxidizing effects, present in the lower airways in the lungs, may protect against infection; while a lack of the agent in nasal and the orbital cavity may increase susceptibility to infection with *M. leprae* bacilli [18]. After over two millennia, still the mystery of leprosy has not been fully solved; yet unfortunate Hansen’s disease patients now are not recognized as sinful and not forced out of society, nor scared off to the margin of the towns in the fear of spreading the horrifying and disfiguring malady.

In conclusion, this communication overview explores the past and the present state of saliva and leprosy to encounter possible mystery and/or wisdom in the ancient art of healing as in the mixture of “sputum and dirt” as reported in biblical times.

## 2. Methods

Current search engines used for this investigation were: PubMed, Google Scholar, CDC, WHO, Rare and neglected diseases, Medscape, Medline, MDLinx, Science and Nature magazines. These databases were explored under one or combination of at least two keywords (saliva; leprosy; Hansen’s disease; biomarkers; dirt; soil; Egypt, etc.). As an example, PubMed, the main search engine source, was explored for keywords and a number of articles were detected as follows: Leprosy (27329), leprosy and soil (70), saliva (33), paucibacillary leprosy (2006), multibacillary leprosy (2986), Egypt, soil, eye (5) and ancient Egypt, black eye (4). A total of 190 related abstracts and 50 articles were carefully scrutinized. Of those, 24 articles were found relevant to be used to prepare this communication.
